# Amplification of the Angiogenic Signal through the Activation of the TSC/mTOR/HIF Axis by the KSHV vGPCR in Kaposi's Sarcoma

**DOI:** 10.1371/journal.pone.0019103

**Published:** 2011-04-29

**Authors:** Bruno C. Jham, Tao Ma, Jiadi Hu, Risa Chaisuparat, Eitan R. Friedman, Pier Paolo Pandolfi, Abraham Schneider, Akrit Sodhi, Silvia Montaner

**Affiliations:** 1 Department of Oncology and Diagnostic Sciences, School of Dentistry, University of Maryland, Baltimore, Maryland, United States of America; 2 Department of Pathology, School of Medicine, University of Maryland, Baltimore, Maryland, United States of America; 3 Greenebaum Cancer Center, University of Maryland, Baltimore, Maryland, United States of America; 4 Cancer Genetics Program, Beth Israel Deaconess Cancer Center, Department of Medicine, Beth Israel Deaconess Medical Center, Harvard Medical School, Boston, Massachusetts, United States of America; 5 Wilmer Eye Institute, Johns Hopkins School of Medicine, Johns Hopkins University, Baltimore, Maryland, United States of America; University of Pennsylvania, United States of America

## Abstract

**Background:**

Kaposi's sarcoma (KS) is a vascular neoplasm characterized by the dysregulated expression of angiogenic and inflammatory cytokines. The driving force of the KS lesion, the KSHV-infected spindle cell, secretes elevated levels of vascular endothelial growth factor (VEGF), essential for KS development. However, the origin of VEGF in this tumor remains unclear.

**Methodology/Principal Findings:**

Here we report that the KSHV G protein-coupled receptor (vGPCR) upregulates VEGF in KS through an intricate paracrine mechanism. The cytokines secreted by the few vGPCR-expressing tumor cells activate in neighboring cells multiple pathways (including AKT, ERK, p38 and IKKβ) that, in turn, converge on TSC1/2, promoting mTOR activation, HIF upregulation, and VEGF secretion. Conditioned media from vGPCR-expressing cells lead to an mTOR-dependent increase in HIF-1α and HIF-2α protein levels and VEGF upregulation. In a mouse allograft model for KS, specific inhibition of the paracrine activation of mTOR in non-vGPCR-expressing cells was sufficient to inhibit HIF upregulation in these cells, and abolished the ability of the vGPCR-expressing cells to promote tumor formation *in vivo*. Similarly, pharmacologic inhibition of HIF in this model blocked VEGF secretion and also lead to tumor regression.

**Conclusions/Significance:**

Our findings provide a compelling explanation for how the few tumor cells expressing vGPCR can contribute to the dramatic amplification of VEGF secretion in KS, and further provide a molecular mechanism for how cytokine dysregulation in KS fuels angiogenesis and tumor development. These data further suggest that activation of HIF by vGPCR may be a vulnerable target for the treatment of patients with KS.

## Introduction

Kaposi's sarcoma (KS) is a multifocal vascular neoplasm invariably associated with infection with the KS-associated human herpesvirus (KSHV/HHV8), which is characterized by cytokine dysregulation [1]. The driving force of the KS lesion is the KSHV-infected spindle-shaped tumor (spindle) cell, thought to have a vascular endothelial or endothelial precursor origin. The promotion of the angiogenic phenotype in these lesions is supposed to be mediated by elevated levels of pro-inflammatory and pro-angiogenic secretions (cytokines, chemokines and growth factors) from the KS spindle cells [1]. Indeed, KS is often thought to result from reactive endothelial hyperproliferation induced by the chronic release of these molecules and has served as a model for tumor- and inflammation-induced angiogenesis [1], [2].

Among the angiogenic factors elaborated by the KS spindle cells, VEGF is unique for its profound impact on KS pathogenesis. VEGF is expressed at elevated levels by KSHV-infected endothelial cells in vitro, and by KS spindle cells in vivo; a strict requirement for VEGF has also been demonstrated for KS spindle cells grown in vitro [3], [4], [5], [6]. These observations are suggestive of an autocrine mechanism for this angiogenic factor in the growth of this vascular tumor. Indeed, like VEGF, its cognate receptor, VEGFR2 (KDR), is also upregulated in AIDS-KS primary lesions as well as in AIDS-KS spindle cell cultures [4], [7], [8]. However, reasonable disagreement remains as to the precise molecular mechanism whereby KSHV promotes VEGF secretion.

Of interest, KSHV ORF74 encodes for a viral G protein-coupled receptor (vGPCR) with close homology to the mammalian CXCR1 and CXCR2 and ligand-independent (constitutive) activity [9], [10]. When expressed upon endothelial-specific retroviral infection or as a transgene, vGPCR is sufficient to recapitulate KS-like lesions in mice [11], [12], [13]. Indeed, emerging data suggest that vGPCR may play a role in KS initiation, progression and maintenance [12], [14], [15].

vGPCR has proven to be a powerful oncogene and a potent angiogenic activator [1]. Expression of vGPCR in endothelial cells activates intracellular pathways that induce cell survival and transformation. In addition, vGPCR-expressing cells elaborate pro-angiogenic factors that are thought to promote the recruitment and transformation of neighboring endothelial cells [16]. Both VEGF and KDR have been previously reported to be upregulated by cells expressing vGPCR [7], [8], [17],[18]. However, the limited expression of this viral oncogene in only a few tumor cells in both transgenic KS mouse models and human KS tissues raises the question as to the relative contribution of vGPCR to VEGF secretion, suggesting that the relationship between vGPCR and VEGF is not clearly established. Here we set out to further characterize the contribution of vGPCR to the upregulation of VEGF secretion in KS.

## Results

### vGPCR activates VEGF expression through a paracrine mechanism dependent on mTOR

**Figure 1:**
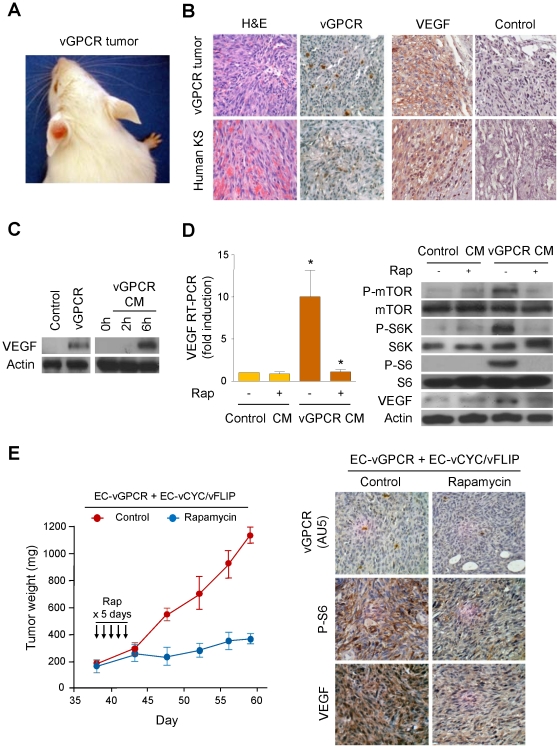
vGPCR activates VEGF expression through an mTOR-dependent paracrine mechanism. (A) KS-like lesion developed upon retroviral transduction of vGPCR in *TIE2*-tva mice (vGPCR tumor), as described in Materials and Methods. (B) H&E and immunohistochemical stainings of vGPCR tumor and human KS, using antibodies against vGPCR, VEGF or an isotype-matched control antibody. (C) Upregulation of VEGF in HMEC1s transfected with Tet REV TA and pBIG AU5 vGPCR and treated with doxycycline (vGPCR), respect to untreated cells (Control). VEGF upregulation in HMEC1 exposed for 2 h or 6 h to supernatants collected from vGPCR-expressing cells (vGPCR CM). (D) Supernatants from vGPCR-expressing cells (vGPCR CM) or control cells (Control CM) were used to treat HMEC1 in the presence or absence of Rapamycin (50 nM). RT-PCR and Western blot analysis were used to determine levels of VEGF mRNA and protein, respectively. (E) EC-vGPCR (10%) were mixed with EC- vCYC/vFLIP (90%), and injected into athymic nu/nu mice for allograft formation. Tumor weight curves, and immunohistochemical detection in tumor tissue of (AU5-tagged) vGPCR-expressing cells, phosphorylated ribosomal S6 protein or VEGF, upon treatment with (10 mg/kg) Rapamycin of vehicle (Control), are shown.

The KSHV-encoded vGPCR causes angioproliferative lesions remarkably similar to human KS, when expressed upon endothelial-specific retroviral infection in immunocompetent animals (Fig. 1A–B) [12]. Interestingly, immunohistochemical staining of these vGPCR tumors using a specific vGPCR antibody reveals the presence of this viral protein in only a small percentage of tumor cells, similar to the expression pattern of vGPCR in human KS, suggestive of a paracrine contribution of vGPCR to Kaposi's sarcomagenesis (Fig. 1B) [12], [13]. In this regard, vGPCR has been implicated in the induction of the expression of VEGF, a key angiogenic factor highly upregulated in KS [17], [18]. However, staining of vGPCR tumors – and human KS – with a specific antibody against VEGF reveals a robust expression of this factor in most tumor cells (Fig. 1B), indicating that vGPCR may also upregulate VEGF in neighboring cells through an indirect mechanism. To explore this possibility, we used an inducible (Tet-on) expression system for vGPCR in immortalized human microdermal endothelial cells (HMEC1). Using this system, we confirmed that induction of vGPCR expression in endothelial cells leads to the potent upregulation of VEGF, as previously reported (Fig. 1C) [17], [18]. Moreover, we observed that exposure of HMEC1 to media conditioned by these vGPCR-expressing cells (vGPCR CM) is similarly able to promote VEGF expression (Fig. 1C).

We therefore set out to determine the mechanism whereby vGPCR angiogenic factors can induce VEGF upregulation. In this regard, we have previously reported that vGPCR paracrine secretions activates TSC/mTOR, a signaling route that has been shown to regulate the expression of VEGF [19], [20]. We thus treated HMEC1s with supernatants derived from vGPCR-expressing cells (vGPCR CM) or control cells (Control CM), in the absence or the presence of the mTOR inhibitor, Rapamycin. Figure 1D shows that exposure of endothelial cells to vGPCR secreted factors leads to the upregulation of the transcription and translation of VEGF in a Rapamycin-sensitive manner.

To further evaluate the contribution of the vGPCR-induced paracrine activation of mTOR to VEGF upregulation *in vivo*, we used an allograft model in which (SV-40) immortalized murine endothelial cells (SVECs) expressing vGPCR (EC-vGPCR) are mixed with SVECs co-expressing two non-tumorigenic KSHV latent genes, vCyclin and vFLIP (EC-vCYC/vFLIP), in a (1∶10) ratio that approximates the proportion of expressing cells in human KS (Fig. 1E) [15]. Cells expressing vCYC and vFLIP do not show VEGF upregulation *in vitro* nor are they tumorigenic *in vivo* (data not shown; [15]). However, these cells (EC-vCYC/vFLIP) are able to induce allografts in nude mice when co-injected with vGPCR-expressing cells (EC-vGPCR) (Fig. 1E). Immunohistochemical staining of these lesions also shows upregulation of VEGF in most tumor cells (Fig. 1E). Interestingly, when these mixed-cell allografts are treated with Rapamycin, which is able to block vGPCR tumorigenesis [19], a reduction in VEGF expression in treated tumors is observed. Collectively, these results suggest that the paracrine upregulation of VEGF by vGPCR requires the activation of the mTOR signaling cascade *in vitro* and *in vivo*.

### vGPCR paracrine secretions activate mTOR through multiple signaling pathways

Diverse extracellular stimuli influence mTOR activity through posttranslational modification of TSC1/TSC2 [20]. Indeed, numerous kinases are able to phosphorylate TSC1 or TSC2, integrating extracellular signals and tumorigenesis through the control of TSC1/TSC2/mTOR [21]. Among these kinases, AKT, ERK, and p38/MK2 have been shown to phosphorylate TSC2 in specific sites, including Thr^1462^, Ser^664^ and Ser^1254^, respectively [22], [23], [24], [25], [26]. Conversely, IKKβ induces mTOR activation by phosphorylating TSC1 in Ser^487^ and Ser^511^ [27].

**Figure 2:**
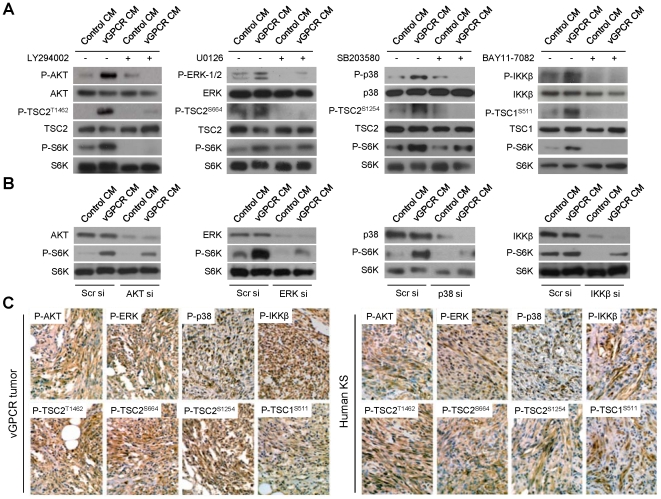
vGPCR secretions regulate TSC/mTOR through multiple signaling pathways *in vitro* and *in vivo*. (A) HMEC1s were pretreated with vehicle or inhibitors of the AKT, ERK, p38 or IKKβ pathways, LY294002 (50 µM), U0126 (50 µM), SB203580 (50 µM) or BAY11-7082 (40 µM). Cells were then exposed to media conditioned by control cells (Control CM) or vGPCR-expressing cells (vGPCR CM). Phosphorylation levels of the corresponding kinase (AKT, ERK1/2, p38 or IKKβ), TSC2/1 targeted phosphorylation site (P-TSC2^T1462^, P-TSC2^S664^, P-TSC2^S1254^ or P-TSC1^S511^), and S6K are shown. (B) HMEC1s were transfected with Scrambled siRNA or siRNA for AKT, ERK (ERK1 and 2), p38 or IKKβ. Cells were then exposed to media conditioned by control or vGPCR-expressing cells. Levels of the corresponding kinase (AKT, ERK1/2, p38 or IKKβ) and S6K are shown. (C) Immunohistochemical staining of vGPCR tumors and human KS with antibodies against P-AKT, P-ERK, P-p38 or P-IKKβ, and the corresponding TSC2/1 targeted phosphorylation site, P-TSC2^T1462^, P-TSC2^S664^, P-TSC2^S1254^ or P-TSC1^S511^.

We have previously shown that induction of AKT activity by vGPCR promotes mTOR activation in neighboring cells [19]. However, the contribution of other kinases upstream of TSC/mTOR to vGPCR oncogenesis remains unclear. We thus treated HMEC1 with media conditioned by control or vGPCR-expressing cells and assessed the activation of TSC kinases by these supernatants. In addition to AKT, we found that ERK, p38 and IKKβ were also activated by vGPCR paracrine secretions (Fig. 2A). Activation of these kinases correlated with the phosphorylation of TSC2 in Thr^1462^, Ser^664^ and Ser^1254^, and phosphorylation of TSC1 in Ser^511^, respectively, and the upregulation of mTOR activity, assessed by S6K phosphorylation (Fig. 2A). We also evaluated the activation of these TSC1/2 kinases by individual GPCR angiogenic factors [14], [28]. Figure S1 shows that all the cytokines tested were able to induce phosphorylation of TSC1 and/or TSC2. Interestingly, IL-1β, IL-10, TNFα and VEGF are each able to promote phosphorylation of TSC1/2 on at least three separate sites (Fig. S1). Collectively, these results suggest that the secreted factors elaborated by vGPCR-expressing cells act together to upregulate multiple intracellular signaling pathways that converge on TSC/mTOR.

To determine the relative contribution of these TSC kinases to the paracrine mTOR activation by vGPCR, we used specific inhibitors of PI3K/AKT, MEK/ERK, p38 or IKKβ. Surprisingly, we observed that either pharmacological inhibition of AKT-mediated phosphorylation of TSC2 or IKKβ-mediated phosphorylation of TSC1 lead to a complete inhibition of mTOR, measured by S6K phosphorylation (Fig. 2A). Conversely, pharmacological inhibition of either ERK or p38 leads to only partial inhibition of ERK-mediated or p38-mediated S6K phosphorylation, respectively. We then used siRNA to specifically knock-down expression of AKT, ERK1 and 2, p38, or IKKβ. Knock-down of these kinases was only sufficient to partially inhibit mTOR (Fig. 2B). Collectively, these results suggest that there is a redundancy in the pathways leading to the phosphorylation of TSC1 and 2 and the activation of mTOR by the paracrine secretions of vGPCR-expressing cells.

We then stained murine vGPCR tumors and human KS tissues with specific antibodies against the phosphorylated (activated) forms of AKT, ERK, p38 or IKKβ or against the corresponding TSC2/TSC1 phosphorylated form, induced by each kinase (Fig. 2C). In support of our in vitro observations, we found phosphorylation of these four kinases and the corresponding targeted aminoacids in TSC2 or TSC1, in both vGPCR tumors and human KS (Fig. 2C). These findings suggest a role for these kinases upstream of TSC/mTOR in vGPCR tumorigenesis and Kaposi's sarcomagenesis *in vivo*.

### Paracrine activation of TSC/mTOR by vGPCR angiogenic factors results in the upregulation of HIF-1α/2α

**Figure 3:**
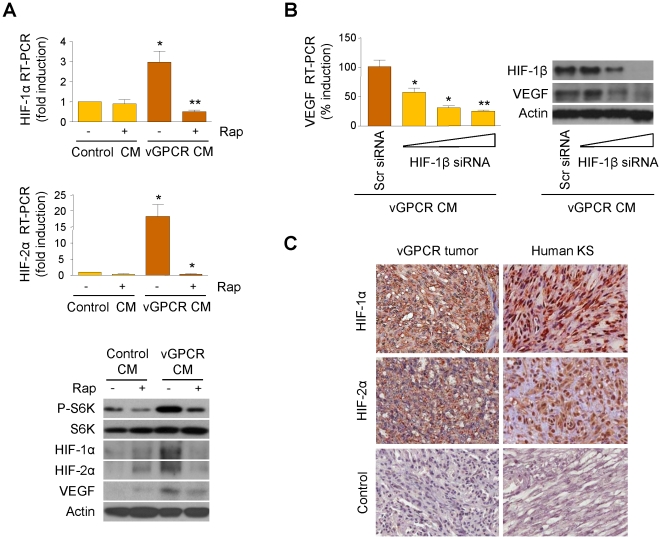
Upregulation of HIF-1α/2α by vGPCR paracrine secretions. (A) HMEC1s were exposed to media conditioned by control (Control CM) or vGPCR-expressing cells (vGPCR CM), in the presence of vehicle or Rapamycin (50 µM). mRNA levels of HIF-1α and HIF-2α and protein levels of HIF-1α, HIF-2α and VEGF are shown. (B) HMEC1s were transfected with increasing doses (20, 40 and 80 nM) of HIF-1β siRNA or Scrambled siRNA. Cells were then exposed to media conditioned by vGPCR-expressing cells. VEGF mRNA levels are shown. (C) Immunohistochemical analysis of HIF-1α and HIF-2α, and staining using an isotype-matched control antibody, in vGPCR tumor and human KS.

TSC/mTOR has been shown to regulate Hypoxia Inducible Factor (HIF), a family of transcription factors containing an inducible α subunit and a constitutive β subunit [20], [29]. HIF promotes neovascularization and vascular remodeling by controlling the expression of key angiogenic proteins, including VEGF [30]. Since vGPCR angiogenic factors activate TSC/mTOR through a variety of intracellular routes, we next investigated whether this activation could lead to the upregulation of VEGF through a HIF-dependent mechanism. To this end, we treated HMEC1 with media conditioned by vGPCR-expressing cells or control cells, in the absence or presence of Rapamycin, and examined the levels of HIF-1α and HIF-2α mRNA and protein. Interestingly, we found that vGPCR angiogenic factors induced an upregulation of HIF-1α mRNA (3-fold) and HIF-2α mRNA (18-fold); both were blocked by the mTOR inhibitor (Fig. 3A). Upregulation of HIF-1α, HIF-2α and VEGF protein levels by vGPCR secretions was also blocked by Rapamycin (Fig. 3A). Furthermore, the increase in VEGF transcription and translation by vGPCR supernatants was blocked by the expression of a specific siRNA of HIF-1β (Fig. 3B). When we investigated the levels of HIF-1α and HIF-2α proteins in vGPCR murine tumors and KS biopsy specimens by immunohistochemical analysis, we found that both vGPCR murine tumors and human KS showed a remarkable overexpression of these transcription factors, compared to normal skin (results not shown) (Fig. 3C). Collectively, these results suggest that vGPCR may induce paracrine upregulation of VEGF through an mTOR/HIF-dependent mechanism.

### Paracrine activation of mTOR is required for HIF upregulation in vGPCR sarcomagenesis *in vivo*

**Figure 4:**
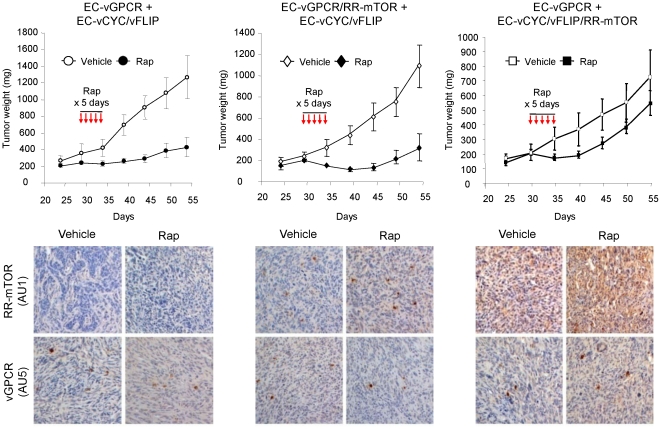
Paracrine activation of mTOR is required for vGPCR sarcomagenesis *in vivo*. Tumor allografts were generated upon injection of athymic nu/nu mice with EC-vGPCR (10%) + EC-vCYC/vFLIP (90%) cells, EC-vGPCR/RR-mTOR (10%) + EC-vCYC/vFLIP (90%) cells or EC-vGPCR (10%) + EC-vCYC/vFLIP/RR-mTOR (90%) cells. Lesions were then treated with (10 mg/kg) Rapamycin or vehicle. Curves of tumor growth and immunohistochemical staining of tumor tissue with anti-AU1 or anti-AU5 antibodies, revealing expression of RR-mTOR or vGPCR, respectively, are shown.

**Figure 5:**
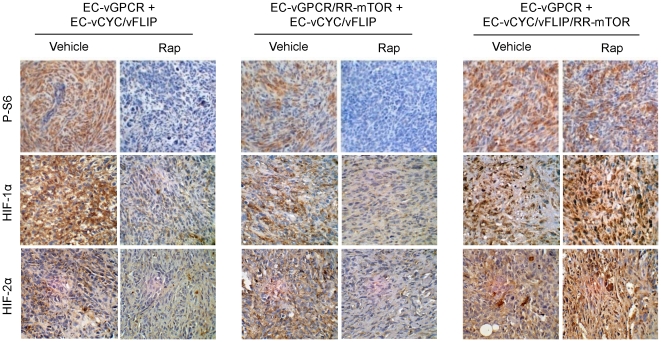
Paracrine activation of mTOR is required for HIF upregulation in vGPCR sarcomagenesis. Immunohistochemical staining with specific antibodies against phospho-S6 ribosomal protein, HIF-1α and HIF-2α of the allografts generated upon injection of EC-vGPCR (10%) + EC-vCYC/vFLIP (90%), EC-vGPCR/RR-mTOR (10%) + EC-vCYC/vFLIP (90%) or EC-vGPCR (10%) + EC-vCYC/vFLIP/RR-mTOR (90%), treated with vehicle or (10 mg/kg) Rapamycin.

vGPCR activates mTOR through both direct and indirect mechanisms and both may thus contribute to endothelial cell transformation and angiogenic dysregulation in KS. To assess the relative contribution of vGPCR direct versus paracrine activation of TSC/mTOR/HIF to vGPCR oncogenesis, we generated cell lines co-expressing vGPCR or vCYC/vFLIP along with a Rapamycin-Resistant mTOR mutant (RR-mTOR) that bears a Ser^2035^→Ile (SI) substitution in the FKBP12-Rapamycin-binding domain (EC-vGPCR/RR-mTOR or EC-vCYC/vFLIP/RR-mTOR) [19]. Expression of RR-mTOR strongly protected vGPCR- and vCYC/vFLIP-expressing cells from the ability of Rapamycin to inhibit mTOR activation *in vitro* (data not shown). We then established mixed-cell allografts injecting athymic nu/nu mice with EC-vGPCR (10%) + EC-vCYC/vFLIP (90%) cells, EC-vGPCR/RR-mTOR (10%) + EC- vCYC/vFLIP (90%) cells or EC-vGPCR (10%) + EC- vCYC/vFLIP/RR-mTOR (90%) cells and treated established tumors with Rapamycin or vehicle (Fig. 4). Similar to EC-vGPCR+EC-vCYC/vFLIP tumors, growth of allografts formed upon injection with EC-vGPCR/RR-mTOR + EC-vCYC/vFLIP was strongly inhibited by treatment with Rapamycin, suggesting that protection from the inhibition of direct mTOR activation within vGPCR-expressing cells was not sufficient to render these tumors sensitive to the drug. Conversely, allografts derived from the injection of EC-vGPCR (10%) + EC- vCYC/vFLIP/RR-mTOR (90%) cells continued growing even upon treatment with Rapamycin, suggesting that the sensitivity to the drug of these allografts is due to the inhibition of the paracrine activation of mTOR in neighboring cells by the angiogenic factors elaborated by vGPCR-expressing cells (Fig. 4). Of interest, tissue staining with a phospho-S6 ribosomal protein specific antibody confirmed the inhibition of mTOR activity in most cells of EC-vGPCR (10%) + EC-vCYC/vFLIP (90%) as well as EC-vGPCR/RR-mTOR (10%) + EC- vCYC/vFLIP (90%) tumors, but not EC-vGPCR (10%) + EC- vCYC/vFLIP/RR-mTOR (90%) allografts, upon Rapamycin treatment (Fig. 5). Similarly, HIF levels were reduced in treated tumors but remained elevated in most tumor cells of EC-vGPCR (10%) + EC- vCYC/vFLIP/RR-mTOR (90%) allografts even after treatment with Rapamycin (Fig. 5). Collectively, these findings support an essential role of vGPCR paracrine secretions in the mTOR-driven promotion of HIF stabilization and VEGF secretion, and in vGPCR tumorigenesis.

### Inhibition of HIF blocks vGPCR tumorigenesis *in vivo*

**Figure 6:**
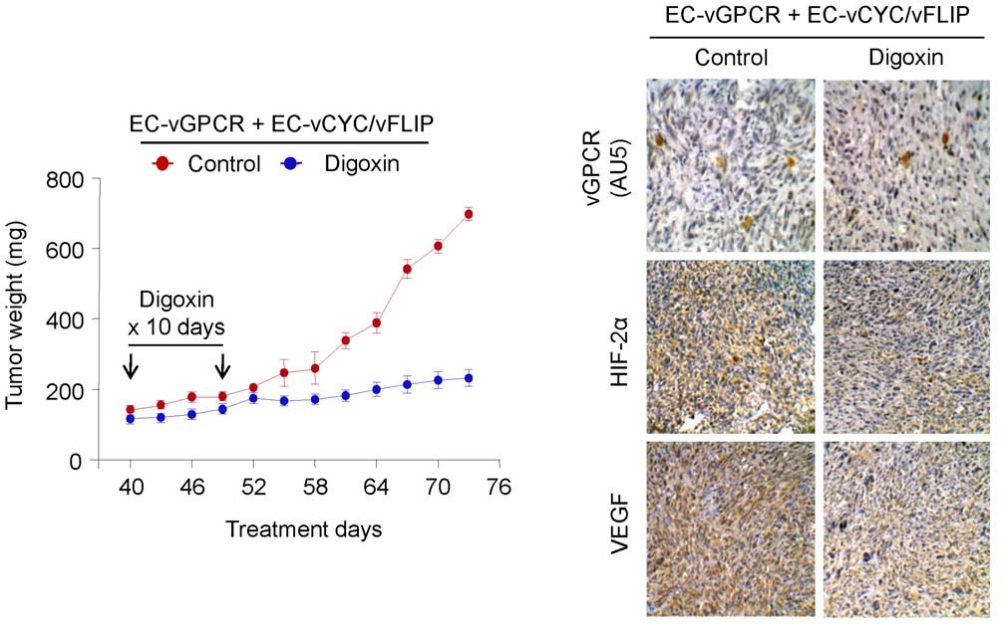
Inhibition of HIF blocks vGPCR tumorigenesis *in vivo.* Tumor allografts were generated upon injection of athymic nu/nu mice with EC-vGPCR (10%) + EC-vCYC/vFLIP (90%) cells. Lesions were treated with (2 mg/kg) Digoxin or vehicle. Tumor growth curves and immunohistochemical staining of tumor tissue with anti-AU5 (revealing vGPCR-expresing cells), anti-HIF-2α or anti-VEGF antibody, are shown.

Of interest, several drugs have been recently described that target HIF expression or activity; these drugs have demonstrated anti-angiogenic and anti-cancer effects *in vivo* [30]. The identification of HIF as a key factor in vGPCR angiogenic amplification prompted us to explore inhibition of HIF as a potential therapeutic approach for KS treatment. We therefore established tumor allografts by injecting mixed-cell populations of EC-vGPCR and EC-vCYC/vFLIP cells in athymic nu/nu mice (Fig. 6). Animals were then treated with either (2 mg/kg) Digoxin, a cardiac glycoside that has been shown to inhibit HIF-1α synthesis and block tumor formation, or vehicle (Control) (Fig. 6) [31]. Drug toxicity, as assessed by weight loss, was minimal in the treated group (reduction <5%) during the treatment period (results not shown). Inhibition of tumor growth by the treatment with Digoxin was sustained for the duration of the experiment. At the end of the study, we observed that the average estimated weight of vehicle-treated tumors was 702 mg (a 4.8 fold increase) vs. an average estimated weight of 234 mg (a 1.7 fold increase) of Digoxin-treated tumors (Fig. 6). Immunohistochemical analysis of these lesions demonstrated a dramatic reduction in the levels of HIF as well as VEGF in the Digoxin-treated animals compared to control mice (Fig. 6). Taken together, our data provide the basis for the early assessment of HIF inhibitors as an anti-KS therapy.

## Discussion

KS is an angioproliferative tumor characterized by the presence of angiogenic and inflammatory mediators [1]. The observation that KS tumors tend to localize to sites of inflammation suggests that these lesions thrive in a cytokine-rich environment [1]. Indeed, KS spindle cells do not appear to be truly transformed; rather, the KS spindle cell elaborates a variety of cytokines, chemokines and growth factors that are essential for their growth and survival. Indeed, isolated KS spindle cells remain strictly dependent on cytokines and growth factors to proliferate in vitro and are not tumorigenic when tested in animal models. Among the numerous angiogenic mediators on which the KS spindle cell is dependent, VEGF has been shown to be essential for KS spindle cell survival in vitro and KS pathogenesis in vivo.

**Figure 7:**
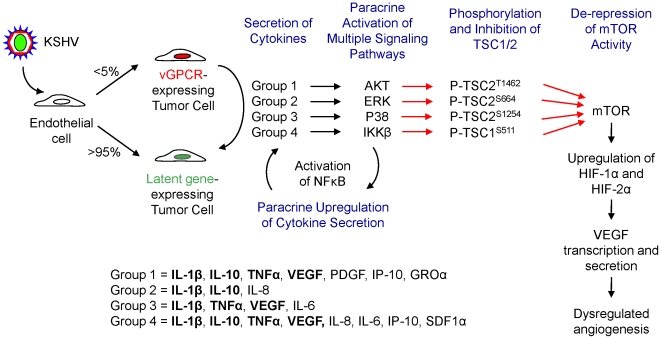
vGPCR cytokines activate VEGF secretion through diverse signaling cascades converging in TSC/mTOR/HIF. Schematic showing the different signaling pathways by which vGPCR cytokines, chemokines and growth factors converge in the phosphorylation of TSC1/2, the activation of mTOR and the upregulation of HIF levels, leading to VEGF secretion.

We previously reported a mechanism whereby the KSHV vGPCR promotes the upregulation of VEGF transcription in vGPCR-expressing cells [17]. We report here that vGPCR also upregulates VEGF through a complex indirect (paracrine) mechanism. Upregulation of VEGF in neighboring (non-vGPCR-expressing) tumor cells results in a dramatic amplification of the angiogenic signal promoted by vGPCR and helps provide an explanation for how this unusual viral oncogene can play a role in KS despite the observation that its expression is restricted to only a few tumor cells. vGPCR angiogenic amplification involves the secretion of angiogenic and inflammatory cytokines by vGPCR-expressing cells which then activate in neighboring cells multiple signaling pathways that ultimately converge on TSC1 and 2, resulting in de-repression of mTOR and the promotion of HIF upregulation and VEGF transcription and secretion (Fig. 7).

TSC kinases activated by vGPCR paracrine secretions include AKT, ERK, p38 and IKKβ, inducing phosphorylation of TSC2/1 at specific regulatory sites [22], [23], [24], [25], [26], [27]. Although pharmacological inhibition of AKT-mediated phosphorylation of TSC2 or IKKβ-mediated phosphorylation of TSC1 lead to a complete inhibition of mTOR activity, siRNA knock down expression of AKT, ERK1 and 2, p38, or IKKβ was only sufficient to partially inhibit S6K phosphorylation. This suggests either that the PI3K/AKT inhibitor, LY294002, and the IKKβ inhibitor, BAY 11-7082, have non-specific inhibitory effects, or that the RNAi knockdown of AKT and IKKβ were less efficient than their pharmacological inhibition. Although we suspect the former to be true, regulation of mTOR through these pathways has proven to be quite complex and the answer may not prove to be straightforward. Nonetheless, our results collectively suggest that there is a redundancy in these pathways and in the phosphorylation sites for inhibiting TSC activity and further provide insight into the complexity of mTOR regulation by different exogenous stimuli.

A number of vGPCR factors promote the phosphorylation of TSC1/2 through several of these signaling pathways. Surprisingly, we demonstrate here that phosphorylation of TSC1 in Ser^511^ by IKKβ is activated by most of the cytokines tested (Fig. S1), suggesting that regulation of mTOR by IKKβ may be quite promiscuous. Moreover, as IKKβ activation leads to the activation of NFκB, this, in turn, may promote a positive feedback loop, further enhancing cytokine – and therefore VEGF – secretion in KS. Of note, paracrine secretions from vGPCR-expressing cells promote a gene expression profile with an NFκB signature in endothelial cells [32]. In light of our results here, this suggests that the NFκB signature may be mediated by the specific secreted cytokines that promote the activation of IKKβ. Ultimately, further investigation into the relative contribution of each of these cytokines to the angiogenic phenotype in KS as well as in other tumors may be warranted.

Several additional KSHV genes have also been shown to upregulate cytokine levels. These viral proteins, including vFLIP, kaposin A, kaposin B, K1 and K15, undoubtedly contribute to the inflammatory milieu observed in KS [33], [34], [35], [36], [37], [38]. As KSHV infection itself has been demonstrated to induce cytokine release [1], the relative contribution of each of the KSHV genes to cytokine dysregulation and the angiogenic phenotype in KS remains to be determined. Moreover, various inflammatory cytokines, including IL-1, TNF-α and interferon-γ (IFN—γ), are increased upon HIV infection, an important cofactor in KS development [39]. Collectively, these findings suggest that cytokine dysregulation and TSC/mTOR/HIF/VEGF activation may be a general mechanism linking KS co-factors, inflammation, and dysregulated angiogenesis in KS.

The mTOR signaling pathway is a key modulator of protein translation and has previously been identified as a positive regulator of HIF and HIF-dependent responses [20], [30]. Oncogenes activating this pathway have been implicated in tumor-induced angiogenesis in other tumors. vGPCR promotion of the paracrine activation of mTOR may play a similar role in the regulation of HIF in KS. Indeed, upregulation of HIF activity has been observed upon KSHV infection of endothelial cells in culture and HIF stabilization has been previously reported in AIDS-KS lesions [40], [41]. Of note, other KSHV genes (e.g. LANA-1 and IRF-3) have also been suggested to play a role in upregulating HIF activity [42], [43], [44]. Given the central role of HIF in VEGF regulation, and the importance of VEGF in KS, it is certainly reasonable to argue that KSHV may encode a redundancy of mechanisms to ensure that this critical endothelial cell growth factor is available in growing KS tumors.

Here, we show that pharmacological inhibition of HIF upregulation by vGPCR is sufficient to inhibit vGPCR oncogenesis. As the master regulator of the hypoxic vascular response, it should not be surprising that HIF plays a central role in Kaposi's sarcomagenesis. HIF drives transcriptional activation of hundreds of genes involved in vascular reactivity, angiogenesis, arteriogenesis, and the recruitment of endothelial precursor cells, all key steps toward the development of KS [45]. Indeed, it is tempting to speculate that recent publications describing KS regression in patients with iatrogenic KS following a switch in their immunosuppressive treatment to the mTOR inhibitor, Sirolimus, may – in part – be due to its effect on decreasing HIF activation [46], [47]. Collectively, our data ultimately provide the basis for the early assessment of drugs inhibiting HIF in those patients with cutaneous and/or systemic KS.

## Materials and Methods

### Expression plasmids and reagents

The expression plasmid encoding for the rapamycin-resistant mTOR mutant (RR-mTOR) that bears a Ser^2035^→Ile (SI) substitution in the FKBP12-rapamycin-binding domain has been described elsewhere [19]. A tetracycline inducible system (Tet-on) was used for vGPCR expression. pCEFL Tet REV TA and pBIG AU5 vGPCR were kindly provided by Dr. Silvio Gutkind (NIDCR, NIH). GRO-α was obtained from R&D Systems; IL-8, VEGF, PDGF, IL-1β, IL-10, IL-6, TNFα, IP-10 and SDF-1α were obtained from Peprotech. Rapamycin, LY294002, U0126, SB203580 and BAY 117082 were purchased from Calbiochem and Digoxin from Sigma. All siRNA oligos were obtained from Qiagen.

### Cell lines, transfections and supernatant collection

Immortalized human dermal microvascular endothelial cells (HMEC1) were obtained from the Centers of Disease Control (Atlanta, GA) and grown in Gibco MCDB 131 medium (Invitrogen), supplemented with 10% FBS, 10 mM/l L-Glutamine, 10 ng/ml epidermal growth factor, 1 µg/ml hydrocortisone and 1% antibiotic antimycotic. (SV-40) immortalized murine endothelial cells (SVEC) and SVEC-derived stable cell lines expressing KSHV vGPCR or KSHV vCyclin and vFLIP (EC-vGPCR, EC-vCYC/vFLIP) were described previously [15]. The Rapamycin-Resistant mTOR (RR-mTOR) was transfected along with the pTracer-EF/Bsd plasmid (Invitrogen) into EC-vGPCR or EC-vCYC/vFLIP. Cells were then stably selected with Blasticidin (Invitrogen). siRNA delivery into cultured cells was performed using Hiperfect (Qiagen). For conditioned media preparation, HMEC1 were transfected with Tet REV TA and pBIG AU5 vGPCR, serum starved and treated with doxycycline (Dox; 1µg/ml). 24 h later, supernatants from untreated (Control CM) and Dox treated (vGPCR CM) cells were collected, centrifuged at 1000 g for 10 min, and concentrated 10 times with centrifugal filters (Millipore).

### Reverse transcriptase PCR

Total RNA was isolated using the GenElute Mammalian Total RNA Miniprep kit (Sigma-Aldrich) according to manufacturer's protocol and reverse transcription was performed using SuperScript III First-Strand Synthesis System (Invitrogen). Upon extraction of mRNA, cDNA was obtained using the SuperScript III First-Strand Synthesis System from Invitrogen. Subsequently, the PCR reaction was carried out using the Mastercycler thermocycler from eppendorfs (2 min at 94°C; 30 cycles of 94°C for 30 seconds, 50°C for 30 seconds, 72°C for 1 minute; and 5 minutes at 72°C). Primers for human VEGF were: 5′-GGGCAGAATCATCACGAAGT-3′ (sense) and 5′-TGGTGATGTTGGACTCCTCA-3′ (anti-sense). Primers for human HIF-1α were: 5′-GCAAGCCCTGAAAGCGCAAG-3′ (sense) and 5′-GTGAGGCTGTCCGACTTTGA-3′ (anti-sense). For HIF-2α, primers employed were: 5′-GTCTCTCCACCCCATGTCTC-3′ (sense) and 5′-GGTTCTTCATCCGTTTCCAC-3′ (anti-sense). Amplification of GAPDH sequence was used for normalization.

### Western blots and Immunohistochemistry

Western blots and immunohistochemistry were performed as previously described [12]. Antibodies recognizing AU1, AU5 and HA epitopes were purchased from Covance. Antibodies against the following proteins were employed: S6K, p-S6K (T389), S6, p-S6 (S240/244), AKT, TSC2, p-TSC2 (T1462), ERK1/2, IKKβ, p-IKK-α/β (S180/181), mTOR, p-mTOR (S2448), p38, p-p38 (T180/Y182), HIF-1α and HIF-1β from Cell Signaling; HIF-2α and P-TSC2 (S664) (immunohistochemistry) from Novus Biologicals; TSC1 from Invitrogen; p-TSC1 (S511) from Bethyl Laboratories; LANA-1 from Leica Microsystems; p-AKT (S473) from R&D Systems; p-ERK1/2 (T202/Y204) from BD Biosciences; and Actin from Santa Cruz. For p-TSC2 (S1254), antibodies from Enogene and Cell Signaling were used for Western blot and immunohistochemistry, respectively. For VEGF, antibodies from R&D Systems and Abcam were used for Western blot and immunohistochemistry, respectively. The antibody recognizing vGPCR was kindly provided by Dr. Gary S. Hayward (Johns Hopkins University, Baltimore, MD).

### Tumorigenesis assays

All procedures involving animals were approved by the Institutional Animal Care and Use Committee. Murine vGPCR tumors were obtained as described in ref 12. Briefly, TIE2-tva transgenic mice expressing in vascular endothelium the avian leukosis virus (ALV) receptor, TVA, were injected i.p. with ALV-derived retrovirus encoding for KSHV vGPCR. Macroscopic vGPCR tumors developed in 4 months predominantly in ear, tail, paw and GI tract. For tumor allograft formation, cells expressing vGPCR (EC-vGPCR or EC-vGPCR/RR-mTOR) were mixed with cells expressing vCyclin and vFLIP (EC-vCYC/vFLIP or EC- vCYC/vFLIP/RR-mTOR), in a (1∶10) ratio (10^5^∶10^6^ cells). These mixed cell populations were then injected into the right flank of 8-wk-old athymic (nu/nu) nude female mice as previously described [15]. For these in vivo studies, Digoxin stock solution (Sigma-Aldrich) was dissolved in DMSO and Rapamycin (LC Laboratories) was dissolved as previously described [19]. Drug treatment was initiated when estimated tumor weight reached around 150–200 mg. For this procedure, tumor-bearing animals were randomly grouped (control, n = 5; drug-treated group, n = 5) and treated with Rapamycin (10 mg/kg) or Digoxin (2 mg/kg) or an equal volume of vehicle. For Rapamycin, treatment schedule was a single injection per animal given i.p. for 5 consecutive days [19]. For Digoxin, treatment schedule was a single injection per animal given i.p. for 10 consecutive days [31]. The animals were monitored twice weekly for tumor formation. The longest length (L) and shortest width (W) of the tumor were measured using a caliper at different time points throughout the experiment. Tumor volume was then converted into tumor weight using the formula LW^2^/2, as described previously [19]. Results of animal experiments were expressed as mean estimated tumor weight ± SD. When appropriate, animals were euthanized, and tissue was fixed in 4% paraformaldehyde and embedded in paraffin for further analysis.

### Human tissues

Four patients diagnosed with KS (multiple lesions) at University of Maryland Dental School or School of Medicine, between 1990 and 2010, were identified. Formalin-fixed paraffin-embedded tissue samples were obtained from the pathology archives for immunohistochemical studies. Representative hematoxylin and eosin sections of each tumor were reviewed and the diagnosis was confirmed by immunohistochemistry using a specific antibody recognizing KSHV Latency-associated Nuclear Antigen-1 (LANA-1). The Institutional Review Board (IRB) protocol was exempt.

### Statistical Analysis

In all cases, results are shown as a mean value ± SD from at least three independent experiments. Western blot scans are representative of at least three independent experiments. Statistical analysis was performed with Prism 4.2 software (GraphPad). ANOVA test: ***, p<0.001; **, p<0.01; *, p<0.05.

## Supporting Information

Figure S1 **Specific vGPCR factors induce TSC2/1 phosphorylation at different sites.** (A) Phosphorylation of S6K and S6 upon treatment of HMEC1 with different vGPCR (recombinant) proteins: IL-8, GROα, VEGF, PDGF, IL-1β, IL-10, IL-6, TNFα, IP-10, or SDF1α. Cells were pretreated with (50 nM) Rapamycin, where corresponding. (B) Phosphorylation of TSC2/1 (TSC2^T1462^, TSC2^S664^, TSC2^S1254^ and TSC1^S511^) or S6K upon treatment of HMEC1 with different recombinant cytokines, chemokines, and growth factors, as described in (A). (C) Analysis of the levels of phosphorylation of TSC2/1 (TSC2^T1462^, TSC2^S664^, TSC2^S1254^ and TSC1^S511^) shown in (B).
(TIFF)
