# Supplementary figures and images for: Amplification of the Angiogenic Signal through the Activation of the TSC/mTOR/HIF Axis by the KSHV vGPCR in Kaposi's Sarcoma
Source: PLoS One. 2011 Apr 29;6(4):e19103. doi: 10.1371/journal.pone.0019103 (PMC3084756; doi:10.1371/journal.pone.0019103)

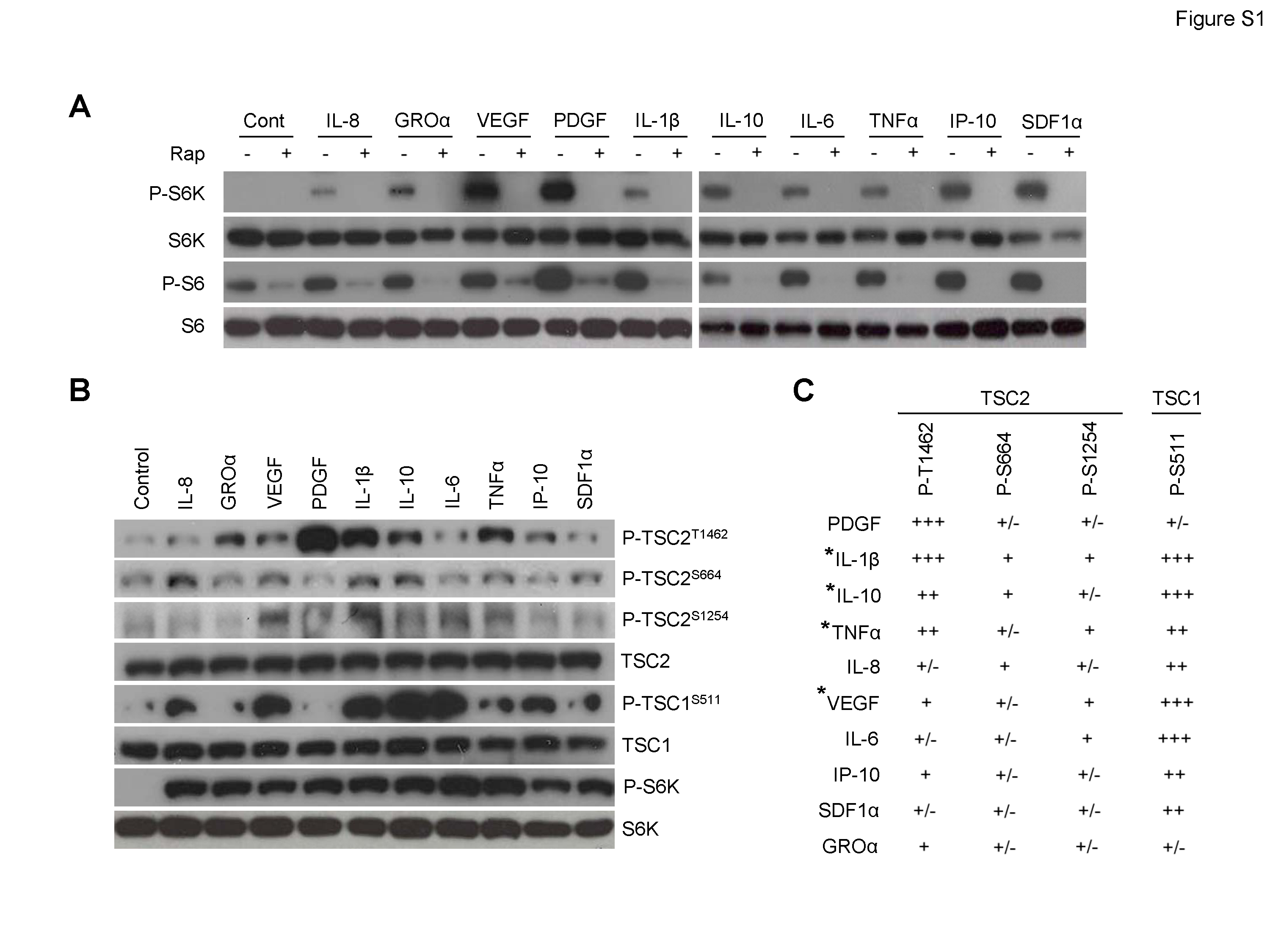

Supplement: Figure S1 — Specific vGPCR factors induce TSC2/1 phosphorylation at different sites. (A) Phosphorylation of S6K and S6 upon treatment of HMEC1 with different vGPCR (recombinant) proteins: IL-8, GROα, VEGF, PDGF, IL-1β, IL-10, IL-6, TNFα, IP-10, or SDF1α. Cells were pretreated with (50 nM) Rapamycin, where corresponding. (B) Phosphorylation of TSC2/1 (TSC2T1462, TSC2S664, TSC2S1254 and TSC1S511) or S6K upon treatment of HMEC1 with different recombinant cytokines, chemokines, and growth factors, as described in (A). (C) Analysis of the levels of phosphorylation of TSC2/1 (TSC2T1462, TSC2S664, TSC2S1254 and TSC1S511) shown in (B). (TIFF) [file pone.0019103.s001.tiff]
